# Arterial wall enhancement indicates higher reperfusion rates in non-ruptured aneurysms after endovascular treatment with stent-assisted coiling

**DOI:** 10.1007/s00234-025-03709-8

**Published:** 2025-07-10

**Authors:** Valentin Ladenhauf, Malik Galijasevic, Milovan Regodic, Verena Rass, Christian Freyschlag, Johannes Deeg, Leonhard Gruber, Michael Swoboda, Jakub Bochnicka, Stephanie Mangesius, Lukas Lenhart, Elke Ruth Gizewski, Astrid Ellen Grams

**Affiliations:** 1 Department of Radiology, Innsbruck Medical University, Innsbruck, Austria; 2 Neuroimaging Research Core Facility, Innsbruck Medical University, Innsbruck, Austria; 3 Neuroimaging Research Core Facility, Innsbruck Medical University, Innsbruck, Austria; 4 Department of Neurology, Innsbruck Medical University, Innsbruck, Austria; 5 Department of Neurosurgery, Innsbruck Medical University, Innsbruck, Austria; 6 Department of Medical Biophysics, Charles University, Prague, Czech Republic

**Keywords:** Aneurysmal wall enhancement, MRI, Aneurysm coiling

## Abstract

**Background and purpose:**

There are differing results in recent literature concerning aneurysmal wall enhancement (AWE) after endovascular treatment (ET) of intracranial aneurysms (IAs). The aim of this retrospective study is to investigate if the presence of AWE of unruptured treated IAs via stent-assisted coiling (SAC) is associated with higher reperfusion rates.

**Materials and methods:**

The clinical courses of 58 patients with IAs after ET via SAC were examined over the timespan of up to 5 years, assessing for AWE in T1 SPACE FS and T1 SE FS blood suppression sequences after contrast administration, events of reperfusion and need for retreatment.

**Results:**

58 patients were included (23 with AWE, 35 without). 18 of 23 patients (78.3%) with AWE showed reperfusion after treatment, compared to 15 of 35 patients (42.9%) without AWE. Reperfusion rates were significantly higher in patients with AWE, compared to those without AWE (*p* = 0.0139) also after propensity score matching (*p* = 0.0456).

**Conclusions:**

In patients with unruptured IAs treated exclusively with SAC, AWE on follow-up MRI was significantly associated with higher reperfusion rates. AWE may serve as an early imaging biomarker of post-treatment instability.

## Introduction

Non-ruptured intracranial aneurysms (IA) are frequently encountered as incidental imaging findings and have a prevalence of 3–5%. However, most patients will not develop clinical symptoms or complications like subarachnoid hemorrhage, which is associated with high morbidity and mortality. The detection of these aneurysms often creates a clinical dilemma for healthcare providers, as they must decide the best course of action to manage the condition effectively.

In routine clinical decision-making, physicians must decide between conservative management and interventional treatments, which consist of surgical or endovascular options. Conservative management is typically achieved via observation, which includes serial imaging studies to monitor the aneurysm over time. This approach aims to avoid unnecessary risks associated with intervention but requires vigilant and continuous follow-up. On the other hand, interventional treatments, which include endovascular procedures such as simple or SAC or surgical approaches, may carry the risk of treatment failure or complications such as ischemia or intracranial hemorrhage [1–3]. The choice between these strategies depends on various factors, including the initial size and growth rate of the aneurysm, patient characteristics, and overall risk assessment [4]. In some aneurysms (e.g. with a neck bigger than 4 mm), endovascular occlusion may only be achieved via SAC [5, 6].

High-resolution vessel wall imaging (HR-VWI) has emerged as the primary dedicated non-invasive imaging tool for the assessment of intracranial aneurysms [7]. HR-VWI aids in the initial decision-making process by providing detailed information about the aneurysm’s wall, helping to identify high-risk aneurysms that might benefit from intervention. Moreover, HR-VWI is vital in posttreatment assessment, usually performed at fixed intervals after endovascular intervention or when any complications are suspected.

One parameter of HR-VWI is the assessment of AWE. AWE has been well established as a pre-treatment risk factor, indicating increased risk of rupture due to wall inflammation or instability [8–10]. Identifying AWE pre-treatment assists in stratifying patients based on their risk and deciding on the urgency and type of intervention required. However, the significance of AWE post-ET remains unclear, with some authors suggesting that it may be linked to flow diversion effects and ongoing inflammatory responses within the aneurysm wall [11] or healing [12], while other authors suggest that it may be linked to reperfusion/recurrence and persisting instability [13, 14].

In this retrospective study, we aimed to investigate if AWE after SAC of unruptured intracranial aneurysms correlates with higher reperfusion rates. The hypothesis was that AWE correlates with an increased risk of aneurysm reperfusion.

By exploring this relationship, we hope to enhance the understanding of post-treatment aneurysm behavior and potentially improve clinical management strategies for patients undergoing endovascular interventions.

## Materials and methods

### Research design

This is a retrospective imaging study, primarily based on the interpretation of MRI and diagnostic subtraction angiography images. It was approved by the research ethics committee of the local university (ECS 1089/2021) and was conducted in compliance with the Declaration of Helsinki. All patients or their legal guardians signed the consent form.

### Patients

Inclusion criteria included the following:

- Age above 18 years.
- A non - ruptured intracranial aneurysm with history of endovascular occlusion via stent - assisted coiling.
- At least two full MRI study with designated sequences for evaluation of arterial wall enhancement (for more details see below).
- At least one diagnostic subtraction angiography in the setting of ET in stent - assisted coiling.

Exclusion criteria included the following:

- History of SAH (prior to treatment or after treatment).
- History of intracranial vascular malformation or fistula.
- History of vasculitis or vessel fibrodysplasia.
- Non diagnostic imaging studies (e.g. motion blurring).
- No sequences for evaluation of AWE (shortened imaging protocol or no administration of contrast agent).

Patient data were queried and extracted from our PACS (Picture Archiving and Communication System) in the time-frame between Jan 01, 2015, and May 31, 2020.

The median clinical follow-up duration was 30 months.

### Image acquisition and analysis

In our facility the MRI images for each patient were acquired using either a 1.5T (Siemens Aera, Siemens Healthcare, Germany) or a 3 T MR imaging scanner (Siemens Skyra, Siemens Healthcare, Germany) with a standard 64-element head coil.

Imaging protocols and specific imaging parameters were standardized in all examined patients and are described in detail in our previous article concerning AWE in IAs [14].

There are two designated sequences in our database, which were used for evaluation of AWE in our study time frame. From 2015 to 2018 our institution used T1 Spin Echo Blood – suppression with fat saturation (T1 SE FS blood suppression). From 2018 on, we switched to T1 sampling perfection with application-optimized contrast using different flip angle evolutions and fat saturation dark blood (T1 SPACE FS db).

Gadolinium was used as a contrast agent and was administered in a typical dose of 0,1 ml/kg of individual body weight.

Ultimately, both sequences readily allow evaluation of intracranial AWE, with the main difference of smaller slice thickness (1 mm vs. 2 mm) in T1 SPACE FS sequences, which is owed to longer acquisition time.

The specific imaging parameters are listed in Table 1.

**Table 1:** Specific sequence parameters for VWI

Sequence	T1 SPACE FS db		T1 SE FS blood-suppression	
Field - Strength	1.5 T	3 T	1.5 T	3 T
Section orientation	Sagittal	Sagittal	Axial	Axial
TR; TE	550ms; 7,2ms	600ms; 8,9ms	641ms; 16ms	709ms; 16ms
FA	120	120	70	70
FOV	248 * 256	220 * 220	220 * 158	220 * 178
Matrix	224 * 256	440 * 440	512 * 368	512 * 416
Slice thickness	1 mm	1 mm	2 mm	2 mm
Scan time	5 min 8 s	6 min 10 s	2 min 5 s	3 min 3 s

### Assessment of intracranial aneurysms in imaging sequences

We assessed the following features and measures:

1. Presence and duration of AWE of the treated aneurysms on MRI.
2. Analysis of aneurysm location on MRI and DAS.
3. Size of aneurysm.
4. Earlier subarachnoid hemorrhage from a bled aneurysm.
5. Aneurysm reperfusion/recanalization.
6. Duration and first appearance of reperfusion.

AWE was defined as visible contrast enhancement of the aneurysm wall on post-contrast T1-weighted sequences (T1 SPACE FS or T1 SE FS), clearly distinguishable from the adjacent vessel lumen and cerebrospinal fluid, assessed visually by an experienced neuroradiologist.

Aneurysm reperfusion was defined as any recurrence of contrast opacification within the aneurysm sac on follow-up MRI or digital subtraction angiography (DSA), following an initially complete occlusion confirmed after SAC.

The number and time interval between MRI studies per patient varies, based on the clinical course (e.g. in patients with visible reperfusion or any complication the time interval was usually shortened). In an uneventful course, MRI is performed in a short timespan after the endovascular intervention (usually not longer than 2 days afterwards) and at 6, 12, 24 and 48 months after ET.

Image interpretation was performed by an experienced neuroradiologist, who was blinded to the written radiological reports. The reports were checked as a second step. In case of differing opinions, a second expert neuroradiologist was consulted.

### Statistical analysis

Statistical analysis was performed using R (R Core Team v. 3.3.0). The normality of data was tested using Kolmogorov-Smirnov and Shapiro-Wilk tests. The normality of data was represented using quantile-plots and histograms. Demographic data was presented using descriptive statistics as mean ± standard deviation (SD) for normally distributed data, and as median [inter-quartile range, IQR] for non - Gaussian distributed data. The statistical differences were calculated using Wilcoxon-Mann-Whitney test. The differences between two groups in reperfusion rates were calculated using Pearson Chi square test with Yates continuity correction and Fisher’s exact test for count data. The data is presented as boxplots. Receiver operating characteristics (ROC) curve were used to analyse diagnostic performance. The cut-off values were determined by using Youden’s J-index. P-values < 0.05 were considered to be statistically significant. In order to balance the data regarding aneurysm size and AWE, we conducted a nearest neighbor propensity score matched analysis. A binary logistic regression analysis was carried out to determine the correlation between the various parameters.

## Results

### General cohort data

58 Patients were included in this retrospective study. 23 patients showed AWE after ET (median age 57 years; 16 female, 7 male. 5 of those 23 patients showed AWE prior to intervention, AWE was persistent in all of them. 35 patients did not show AWE after ET (median age 56a; 26 female, 9 male (Table 2). Demographics showed no significant differences group-wise.

**Table 2:** General cohort data

	Overall sample (*n* = 58)	AWE (*n* = 23)	No AWE (*n* = 35)	*p*-value
Age (years) [IQR, 25–75]	56 [48–62]	57 [49–61]	56 [49 to 61]	0.62
Gender (m/w)	20/46	7/16	9/26	0.76

### Aneurysmal wall enhancement and reperfusion

In the group with AWE (group 1), 18 of 23 (78.3%) patients sustained reperfusion after ET. Of the latter group, 13 patients (41.9%) needed a second ET; 5 patients (16.1%) needed a third ET.

5 of 23 (21.7%) patients already showed AWE prior to any intervention, which was persistent in all 5 cases. The median time difference between first occurrence of AWE and the date of the endovascular intervention was 2 months [1st quartile = 0 months, which means AWE was recorded at the initial post treatment MRI/3rd quartile 25 months].

In 15 patients the occurrence of reperfusion after ET and the first occurrence of AWE was recorded at the same time [median difference 0 months], 9 patients showed AWE prior to reperfusion while 7 patients showed AWE after reperfusion was first present.

In the group without AWE, 15 of 35 (42.9%) patients sustained reperfusion after ET. 6 of 35 (17.1%) needed a second ET, 1 patient (2.9%) needed a third ET.

**Fig. 1:**
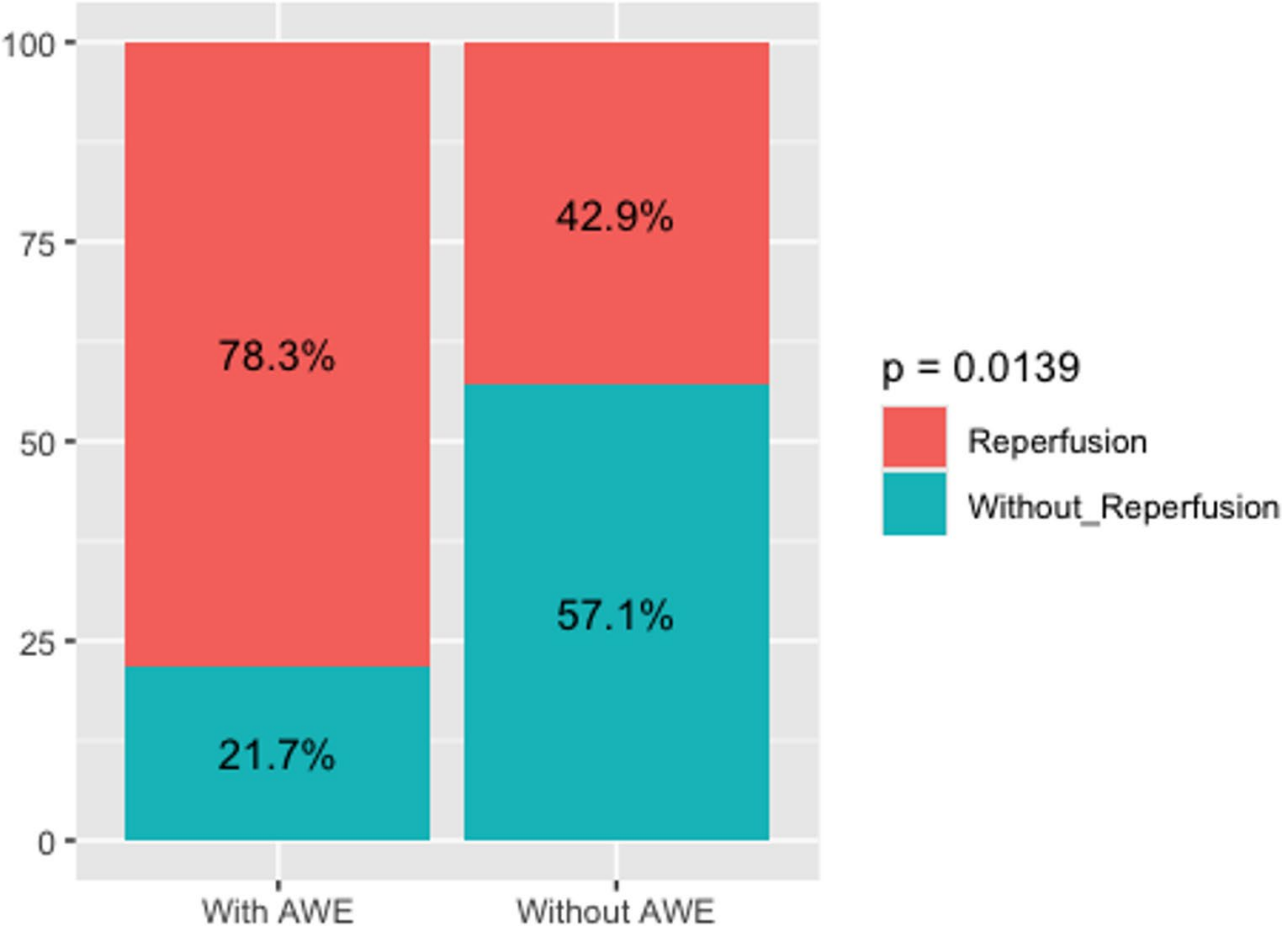
Reperfusion percentage in two patient groups concerning AWE, showing a significant [*p* = 0.0139] difference in aneurysm reperfusion in patients with and without AWE

**Fig. 2:**
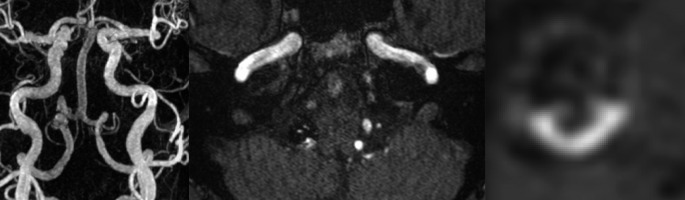
Reperfusion in combination with AWE after treatment via stent - assisted coiling of a right vertebral artery aneurysm. Far left: Contrast enhanced magnetic resonance angiography as a maximum intensity projection in the coronal plane depicting substantial reperfusion of the treated aneurysm. Middle: Intracranial time of flight angiography in an axial plane depicting reperfusion of the treated aneurysm as well. Far right: Heavily zoomed in T1 weighted fs db space sequences after contrast administration in an axial plane depicting semilunar dorsal AWE of the treated aneurysm

In 51 of 58 patients (88.0%) the stent - application occurred in the initial endovascular intervention. In 7 of 58 patients (12.0%) the stent application occurred in a secondary endovascular intervention. 3 of those 7 patients (42.9%) showed AWE after the first intervention, AWE was persistent in all of them after the secondary stent - application (Figs. 1 and 2).

### Aneurysm diameter

**Fig. 3:**
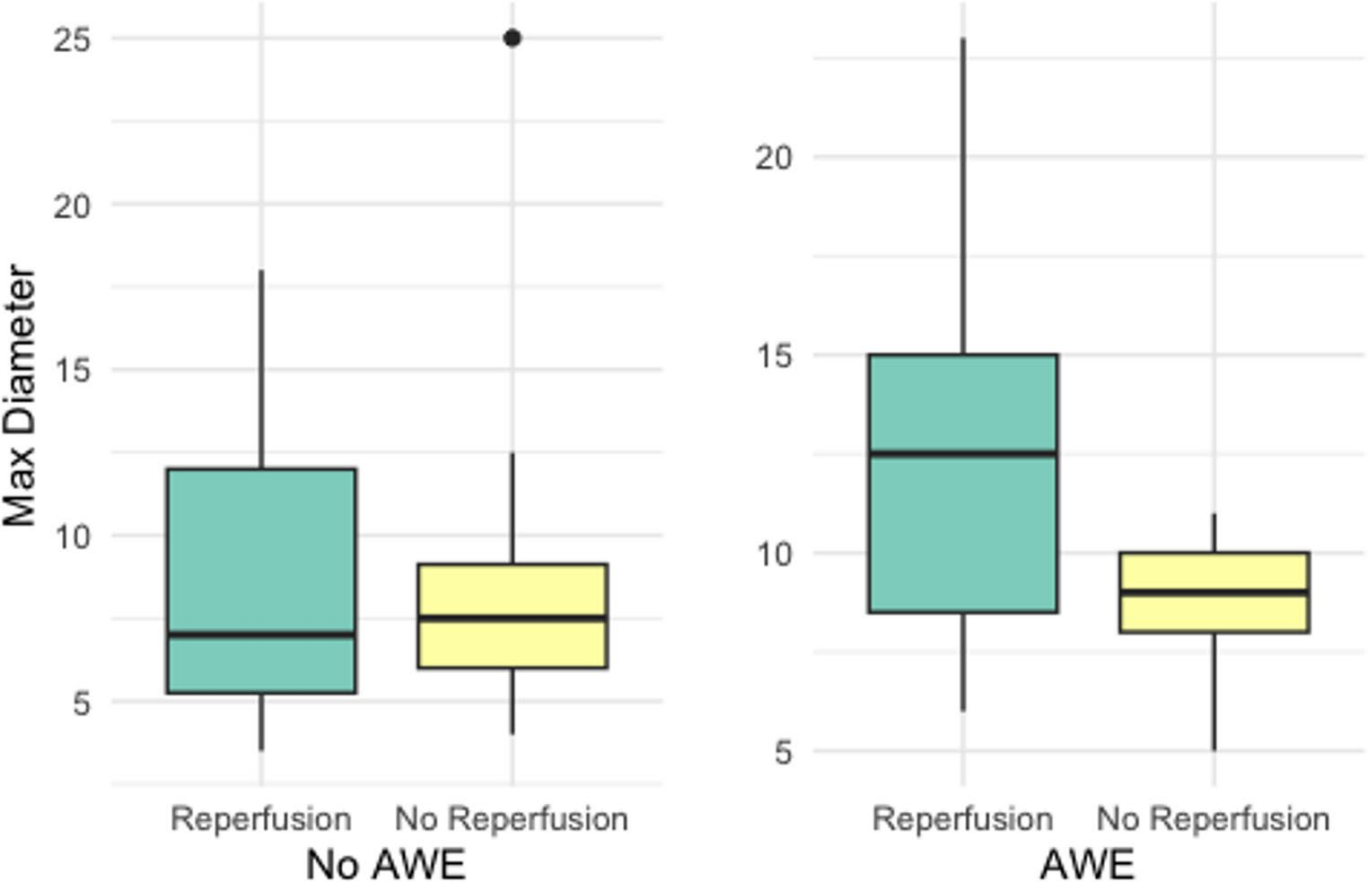
Maximum intracranial aneurysm diameter in patients with and without AWE, concerning reperfusion

The maximum diameter of the intracranial aneurysms with AWE was significantly higher than those without AWE (*p* = 0.0033). There were no significant differences between aneurysms with and without reperfusion (*p* = 0.9), as visually depicted in the following Figure (Fig. 3).

### ROC analysis

**Fig. 4:**
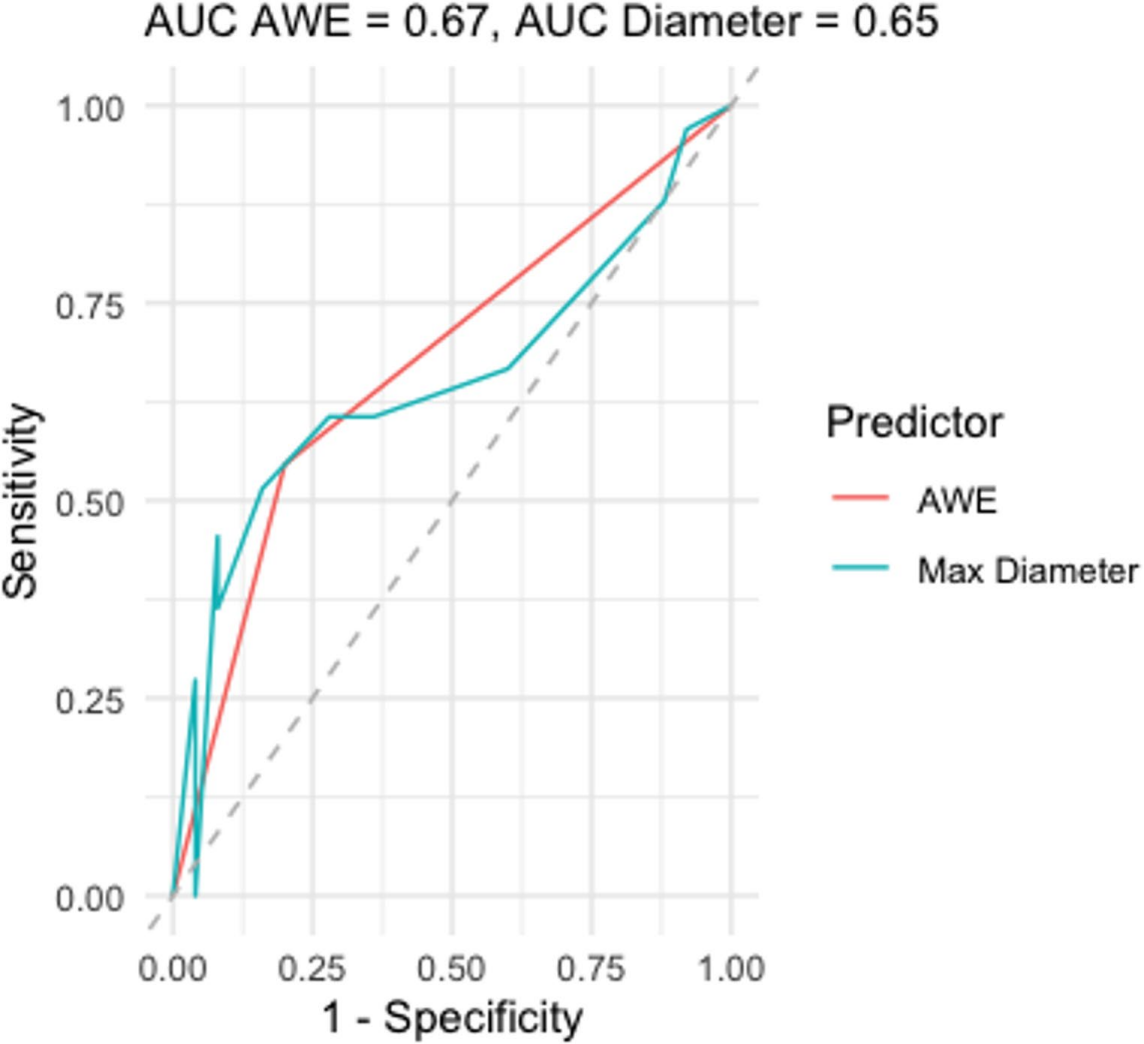
ROC analysis of various parameters vs. aneurysm reperfusion

The ROC curve analysis results (Fig. 4.) concerning reperfusion and various parameters indicates that AWE and the maximum aneurysm diameter are not complete biomarkers for reperfusion with an AUC of 0.67 for AWE and also 0.67 for maximum aneurysm diameter.

AWE as a sole predictor has significantly higher sensitivity (78.3%) compared to maximum aneurysm size (45.5%) but mediocre specificity (57.1%) compared to maximum aneurysm size (92.0%).

### Propensity score matched analysis

**Fig. 5:**
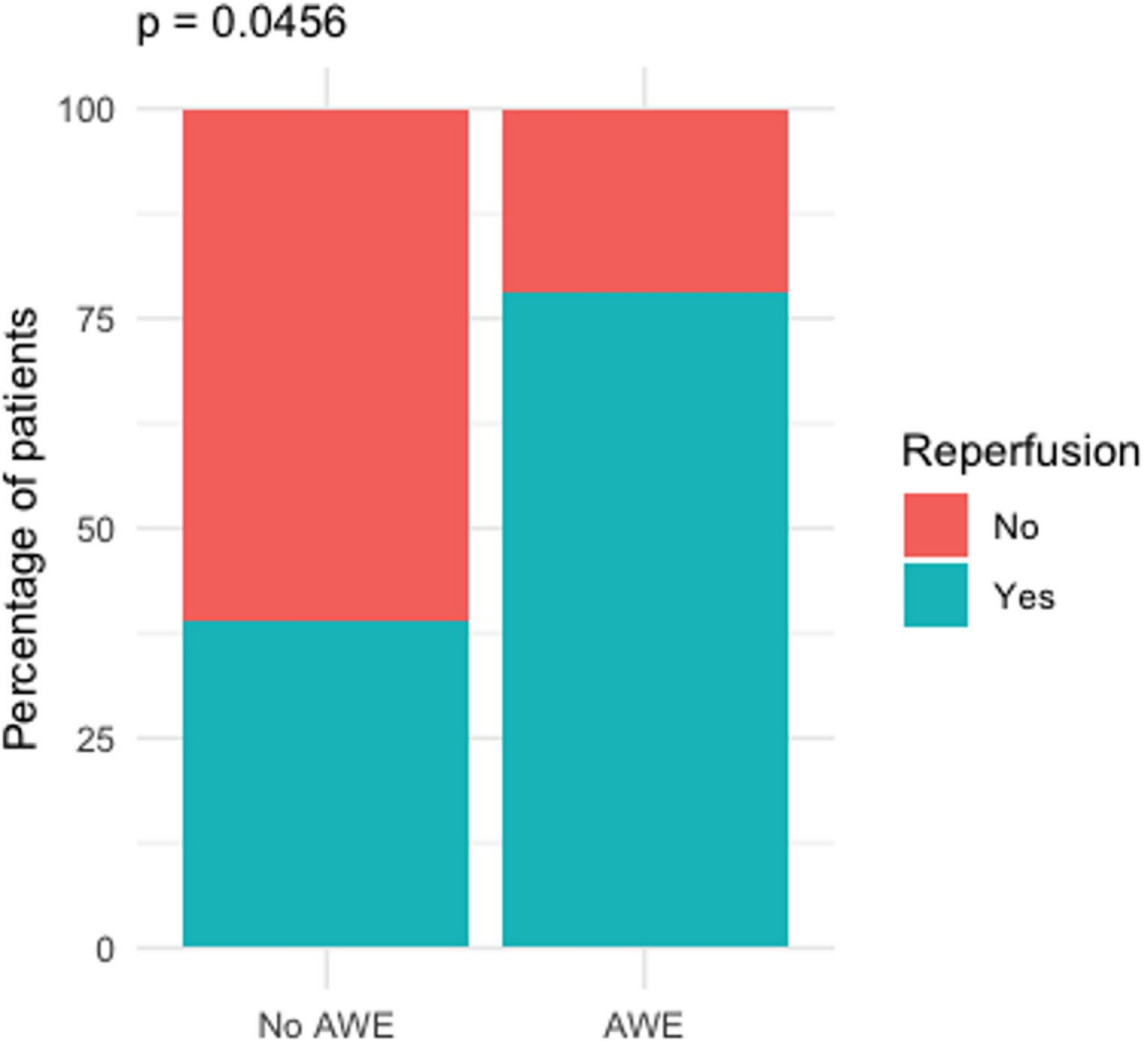
AWE vs. reperfusion in propensity score matched data regarding aneurysm size

To control for aneurysm size, we performed a propensity score matching (PSM) analysis using nearest neighbor matching with aneurysm maximum diameter as the matching variable. A total of 23 patients with AWE were successfully matched to 23 patients without AWE, resulting in a balanced cohort of 46 patients. Matching achieved a substantial reduction in standardized mean difference for aneurysm diameter from 0.64 before matching to 0.29 after matching, with improved variance ratio (from 1.30 to 1.27) and reduced empirical cumulative distribution function (eCDF) distance. After matching, reperfusion occurred in 78.3% (18/23) of patients with AWE and in a smaller proportion of patients without AWE, consistent with the unmatched analysis. These findings underscore the independent association between AWE and aneurysm reperfusion (*p* = 0.0456), even after controlling for aneurysm size (Fig. 5).

### Binary logistic regression analysis

A binary logistic regression analysis (Table 3) was carried out to determine the correlation between reperfusion and sex, age, primary stent application, maximum aneurysm diameter, localization (vessel of origin) and AWE. Results include B-value (estimate), standard error, z-value, 95% confidence intervals (CI), odds ratio (OR) and p-value. The results clearly show a significant (*p* = 0.0216) correlation between reperfusion and AWE.

**Table 3:** Binary logistic regression analysis

*p* - value	Estimate	Std. Error	z-value	95% CI	OR
Sex [male] 0.11	0.56	0.85	0.65	−0.85 to 2.01	1.75
Age [years] 0.22	0.04	0.036	1.2	−0.01 to 0.10	1.04
Primary stent [yes] 0.87	−0.17	1.04	−0.16	−2.03 to 1.49	0.85
Max. diameter [mm] 0.71	0.029	0.076	0.38	−0.10 to 0.16	1.03
Location [reference: basilar artery]					
Vertebral artery 0.87	−0.31	1.86	−0.17	−3.52 to 2.80	0.74
AcomA 0.87	−0.31	1.86	−0.17	−3.52 to 2.80	0.74
PCOM 1.00	−17.36	2399.55	−0.01	n/a	2.91 x e − 8
ACI 0.1198	1.28067	0.82336	1.555	−0.05 to 2.69	3.59904
ACA 0.9948	15.72058	2399.5448	0.007	n/a	0.6719903
ACM 0.97	0.07	1.89	0.04	−3.23 to 3.23	1.072
ACP 0.45	1.10	1.45	0.76	−1.20 to 3.81	3.00
AWE [yes] **0.0216**	1.76	0.77	2.30	0.56 to 3.12	5.82

## Discussion

Our results show that AWE after ET with SAC is significantly associated with aneurysm reperfusion. This finding remained consistent across both unmatched and propensity-matched analyses, and was confirmed by logistic regression.

To account for the fact that aneurysms with AWE tended to be larger, we performed a propensity score matching based on aneurysm diameter. Even after controlling for size, aneurysms with AWE still showed significantly higher reperfusion rates, reinforcing the independent association between AWE and recurrence.

Other clinical or morphological variables, including aneurysm size and location, did not significantly correlate with reperfusion. While AWE demonstrated moderate diagnostic accuracy (AUC 0.67), its high sensitivity suggests that it may be useful as a screening tool to identify patients at risk for reperfusion, particularly when combined with other clinical parameters.

The ROC - analysis concerning reperfusion and AWE showed high sensitivity (78.3%) but low specificity (57.1%), underscoring that AWE as a single predictor is an incomplete biomarker. However, in a real life diagnostic setting this is a negligible issue as vascular assessment is always based on a multifactorial analysis.

Prior studies have reported conflicting results regarding the significance of AWE after ET. Some authors suggest that in ET with stent-implantation, AWE might be due to flow diversion effects [15]. Nikoubashman et al. found nearly universal enhancement (97%) after coiling and suggested limited predictive value [16]. Larsen et al. linked enhancement to healing rather than recurrence, but their cohort included only WEB-device-treated ruptured aneurysms [12]. In contrast, our study focuses exclusively on unruptured aneurysms treated with SAC and suggests that wall enhancement may indicate residual instability and risk of reperfusion.

Interestingly, our data and statistical results suggest otherwise but are based on fundamentally different parameters (arterial wall vs. aneurysm cavity, ET technique and cohort), so the level of comparability is unclear.

A recent study by Leber et al. [13] could show that AWE after ET is associated with aneurysm recanalization/reperfusion, which is similar to the results of this study. However, their patient cohort also included patients who had initially suffered from aneurysm rupture (although their study suggests that aneurysm rupture does not affect AWE) and explicitly excludes patients who received treatment via stent - assisted coiling, which is in contrast to our study design.

This study is limited by its retrospective design, which led to heterogeneity in follow-up intervals, scanner field strength (1.5 T vs. 3 T), and use of two different MRI sequences (T1 SE FS blood suppression and T1 SPACE DS dark blood). While these reflect real-world clinical practice, they introduce variability. Additionally, the assessment of AWE was based on subjective expert interpretation, which may limit reproducibility.

To our knowledge, no study has shown differences in AWE assessment between field strengths. Roa et al. demonstrated no significant differences between 3 T and 7 T MRI [17]. Additionally, Dier et al. recently showed substantial inter-reader variability in subjective AWE assessment, highlighting a limitation of qualitative evaluation [18].

Despite these limitations, our findings suggest that AWE on follow-up MRI after stent-assisted coiling may serve as an early marker of aneurysm reperfusion. Incorporating AWE into routine post-treatment imaging assessments could support early identification of patients who may require closer monitoring or retreatment.

In general, the discussion of AWE after the treatment of intracranial aneurysms remains controversial and indecisive, although recent studies link it to reperfusion of intracranial aneurysms.

## Conclusions

AWE after ET of IA via SAC is a common finding of unclear relevance. In this study, we could show that it is associated with higher reperfusion rates, implying that it might be a marker of instability after ET via SAC. Based on our results, it might be feasible to shorten the interval for follow-up scans of patients who demonstrate AWE after ET via SAC, to quickly filter out patients with reperfusion of treated IA and the need for retreatment.

## Data Availability

The anonymised data can be made available upon request.

## References

[1] Vlak MH, Algra A, Brandenburg R, Rinkel GJ (2011) Prevalence of unruptured intracranial aneurysms, with emphasis on sex, age, comorbidity, country, and time period: a systematic review and meta-analysis. Lancet Neurol 10(7):626–636 21641282 10.1016/S1474-4422(11)70109-0

[2] Etminan N, Rinkel G (2016) Unruptured intracranial aneurysms: development, rupture and preventive management. Nat Reviews Neurol 12:11 10.1038/nrneurol.2016.150 27808265

[3] Morita A, Kirino T, Hashi K, Aoki N, Fukuhara S, Hashimoto N, Takeo N, Sakai M, Teramoto A, Tominari S, Yoshimoto T (2012) The natural course of unruptured cerebral aneurysms in a Japanese cohort. N Engl J Med 366:2474–2482 10.1056/NEJMoa1113260 22738097

[4] Etminan N, de Sousa DA, Tiseo C, Bourcier R, Desal H, Lindgren A, Koivisto T, Netuka D, Peschillo S, L´emeret S et al (2022) European stroke organisation (eso) guidelines on management of unruptured intracranial aneurysms. Eur Stroke J 7(3):LXXXI–CVI 10.1177/23969873221099736 PMC9446328 36082246

[5] Raymond J, Darsaut T (2011) Stenting for intracranial aneurysms: how to paint oneself into the proverbial corner. Am J Neuroradiol 32(9):1711–1713 21903913 10.3174/ajnr.A2700 PMC7965363

[6] Phan K, Huo YR, Jia F, Phan S, Rao PJ, Mobbs RJ, Mortimer AM (2016) Meta-analysis of stent-assisted coiling versus coiling-only for the treatment of intracranial aneurysms. J Clin Neurosci 31:15–22 27344091 10.1016/j.jocn.2016.01.035

[7] Texakalidis P, Hilditch C, Lehman V, Lanzino G, Pereira V, Brinjikji W (2018) Vessel wall imaging of intracranial aneurysms: systematic review and meta-analysis. World Neurosurg 117:06 10.1016/j.wneu.2018.06.008 29902602

[8] Vergouwen MD, Backes D, Van Der Schaaf I, Hendrikse J, Kleinloog R, Algra A, Rinkel G (2019) Gadolinium enhancement of the aneurysm wall in unruptured intracranial aneurysms is associated with an increased risk of aneurysm instability: a follow-up study. Am J Neuroradiol 40(7):1112–1116 31221634 10.3174/ajnr.A6105 PMC7048551

[9] Hartman JB, Watase H, Sun J, Hippe DS, Kim L, Levitt M, Sekhar L, Balu N, Hatsukami T, Yuan C et al (2019) Intracranial aneurysms at higher clinical risk for rupture demonstrate increased wall enhancement and thinning on multicontrast 3d vessel wall mri. Br J Radiol 92(1096):20180950 30653339 10.1259/bjr.20180950 PMC6540871

[10] Gariel F, Ben Hassen W, Boulouis G, Bourcier R, Trystram D, Legrand L, Rodriguez-Regent C, Saloner D, Oppenheim C, Naggara O et al (2020) Increased wall enhancement during follow-up as a predictor of subsequent aneurysmal growth. Stroke 51(6):1868–1872 32397927 10.1161/STROKEAHA.119.028431

[11] Elsheikh S, Urbach H, Meckel S (2020) Contrast enhancement of intracranial aneurysms on 3t 3d black-blood mri and its relationship to aneurysm recurrence following endovascular treatment. Am J Neuroradiol 41(3):495–500 32054618 10.3174/ajnr.A6440 PMC7077883

[12] Larsen N, Fl¨uh C, Madjidyar J, Synowitz M, Jansen O, Wodarg F (2020) Visualization of aneurysm healing: enhancement patterns and reperfusion in intracranial aneurysms after embolization on 3t vessel wall mri. Clin Neuroradiol 30:811–815 31754758 10.1007/s00062-019-00854-5

[13] Leber SL, Hassler EM, Michenthaler M, Renner W, Deutschmann H, Reishofer G (2024) Wall enhancement of coiled intracranial aneurysms is associated with aneurysm recanalization: A cross-sectional study. Am J Neuroradiol 45(5):599–604 10.3174/ajnr.A8174 PMC11288544 38548301

[14] Ladenhauf V, Galijasevic M, Regodic M, Helbok R, Rass V, Freyschlag C, Petr O, Deeg J, Gruber L, Mangesius S et al (2024) Aneurysmal wall enhancement of non-ruptured intracranial aneurysms after endovascular treatment correlates with higher aneurysm reperfusion rates, but only in large aneurysms. Diagnostics 14(14):1533 39061670 10.3390/diagnostics14141533 PMC11276124

[15] Zwarzany L, Owsiak M, Tyburski E, Poncyljusz W (2022) High-resolution vessel wall mri of endovascularly treated intracranial aneurysms. Tomography 8(1):303–315 35202190 10.3390/tomography8010025 PMC8874437

[16] Nikoubashman O, Tabrizi CM, Münstermann M, Schubert GA, Reich A, Wiesmann M, Müller M (2020) Findings and prognostic value of contrast-enhanced early magnetic resonance imaging after coil embolization of cerebral aneurysms. World Neurosurg 135:e382–e385 31816454 10.1016/j.wneu.2019.11.173

[17] Roa JA, Zanaty M, Osorno-Cruz C, Ishii D, Bathla G, Ortega Gutierrez S, Hasan DM, Samaniego EA (2020) Objective quantification of contrast enhancement of unruptured intracranial aneurysms: a high-resolution vessel wall imaging validation study. J Neurosurg 134(3):862–869 32032948 10.3171/2019.12.JNS192746 PMC7415549

[18] Dier C, Justin K, Alhajahjeh S, Sanchez S, Wendt L, Avalos F, Sagues E, Gudino A, Molina D, Shenoy N et al (2025) There is poor agreement between the subjective and quantitative adjudication of aneurysm wall enhancement. Am J Neuroradiol 46(4):689–697 39317479 10.3174/ajnr.A8508 PMC11979857

